# Enhanced catalytic performance of penicillin G acylase by covalent immobilization onto functionally-modified magnetic Ni_0.4_Cu_0.5_Zn_0.1_Fe_2_O_4_ nanoparticles

**DOI:** 10.1371/journal.pone.0297149

**Published:** 2024-01-19

**Authors:** Zhixiang Lv, Zhou Wang, Shaobo Wu, Xiang Yu

**Affiliations:** 1 The People’s Hospital of Danyang, Affiliated Danyang Hospital of Nantong University, Zhenjiang, 212300, P.R. China; 2 Vanadium and Titanium Resource Comprehensive Utilization Key Laboratory of Sichuan Province, College of Vanadium and Titanium, Panzhihua University, Panzhihua, 617000, P.R. China; 3 Zhenjiang Hospital of Chinese Traditional and Western Medicine, Zhenjiang, 212013, P.R. China; Universita degli Studi di Pavia, ITALY

## Abstract

With the emergence of penicillin resistance, the development of novel antibiotics has become an urgent necessity. Semi-synthetic penicillin has emerged as a promising alternative to traditional penicillin. The demand for the crucial intermediate, 6-aminopicillanic acid (6-APA), is on the rise. Enzyme catalysis is the primary method employed for its production. However, due to certain limitations, the strategy of enzyme immobilization has also gained prominence. The magnetic Ni_0.4_Cu_0.5_Zn_0.1_Fe_2_O_4_ nanoparticles were successfully prepared by a rapid-combustion method. Sodium silicate was used to modify the surface of the Ni_0.4_Cu_0.5_Zn_0.1_Fe_2_O_4_ nanoparticles to obtain silica-coated nanoparticles (Ni_0.4_Cu_0.5_Zn_0.1_Fe_2_O_4_-SiO_2_). Subsequently, in order to better crosslink PGA, the nanoparticles were modified again with glutaraldehyde to obtain glutaraldehyde crosslinked Ni_0.4_Cu_0.5_Zn_0.1_Fe_2_O_4_-SiO_2_-GA nanoparticles which could immobilize the PGA. The structure of the PGA protein was analyzed by the PyMol program and the immobilization strategy was determined. The conditions of PGA immobilization were investigated, including immobilization time and PGA concentration. Finally, the enzymological properties of the immobilized and free PGA were compared. The optimum catalytic pH of immobilized and free PGA was 8.0, and the optimum catalytic temperature of immobilized PGA was 50°C, 5°C higher than that of free PGA. Immobilized PGA in a certain pH and temperature range showed better catalytic stability. V_max_ and K_m_ of immobilized PGA were 0.3727 μmol·min^-1^ and 0.0436 mol·L^-1^, and the corresponding free PGA were 0.7325 μmol·min^-1^ and 0.0227 mol·L^-1^. After five cycles, the immobilized enzyme activity was still higher than 25%.

## Introduction

Currently, antibiotics continue to dominate a significant market, encompassing human drugs [1], veterinary medications [2], and pesticides [3,4]. The demand for antibiotics used in conventional applications remains substantial. However, the misuse of antibiotics has given rise to a host of resistance issues [5,6]. In response, the public is actively exploring alternatives to antibiotics [7,8]. Nevertheless, until such alternatives are identified, antibiotics will maintain their pivotal role. Antibiotics are presently synthesized through various methods, including fermentation [9], chemical synthesis [10,11], and enzymatic (semi-synthetic) methods [12]. The fermentation method is susceptible to the influence of various factors, impacting the quality of the process, making product separation challenging, and often resulting in lower purity. Conversely, total synthesis methods, while ensuring high product purity, tend to be costly and unsuitable for large-scale industrial production. In contrast, enzymatic (semi-synthetic) approaches are favored due to the efficiency and specificity of enzymatic reactions. The products from these methods are easier to separate and boast higher purity. Furthermore, in response to limitations associated with traditional antibiotics, derivatives of semi-synthetic antibiotics have the potential to enhance efficacy and mitigate resistance issues [13–15].

Penicillin G acylase (PGA), a commonly employed industrial enzyme, serves four crucial roles, including the resolution of racemic mixtures [16], peptide synthesis [17], the production of semi-synthetic beta-lactam antibiotics [18], and the creation of intermediates for semi-synthetic antibiotics [19]. The 6-aminopicillanic acid (6-APA) itself exhibits minimal antibacterial activity and cannot be directly utilized in clinical applications. However, by utilizing it as a starting material for chemical modification, including the attachment of diverse side chains, a range of semi-synthetic penicillins with potent antibacterial properties, enhanced disease resistance, and convenient administration can be generated [20–22]. It’s worth noting that the chemical synthesis of 6-APA is not only expensive but also involves the use of toxic chemicals [23], while the PGA-mediated catalytic reaction is both environmentally friendly and efficient [24].

PGA is capable of catalyzing the transformation of penicillin G potassium into 6-aminopicillanic acid (6-APA) [25]. However, the application of free PGA in industrial processes is hindered by several disadvantages, including poor stability, rapid inactivation in natural environments, vulnerability, and a lack of long-term stability and recyclability [26,27]. Fortunately, these challenges can be effectively addressed through the use of immobilized PGA [28,29]. Immobilized PGA exhibits a longer half-life and higher stability, offering a significant advantage in terms of reusability, which, in turn, enhances cost-efficiency within the industrial context. Furthermore, immobilized PGA serves the important function of preventing substrate contamination by the enzyme itself or other compounds, resulting in the production of high-purity products [30]. It’s worth noting that although enzyme activity is sometimes compromised due to conformational changes in biomolecular structure and limitations related to mass transfer during immobilization [31], the overall benefits far outweigh these limitations, positioning immobilized PGA as a prominent player in the field of biology [32–34] The carriers for immobilized PGA can be categorized into organic and inorganic materials. Organic materials, such as cellulose [35,36], glucan [37], agarose [38,39], and chitosan [40], offer benefits like wide availability, environmental friendliness, and renewability. However, using natural polymers as carriers often necessitates pre-activation, complicating the synthesis process. Synthetic polymer resin carriers, known for their robust mechanical properties and controllable functional groups [41,42], are another option. Nonetheless, many synthetic macromolecular resin carriers used for immobilized PGA still exhibit certain drawbacks [43]. Some have fine fibers that are challenging to separate from substrates and products, significantly impacting their reusability. Others impede substrate and product diffusion, leading to slower reactions [25]. In contrast to organic carriers, inorganic carriers offer several advantages, including superior corrosion resistance, heat resistance, mechanical strength, large specific surface area, uniform pore size distribution, cost-effectiveness, and improved reusability [44–47]. Additionally, they possess the ability to withstand microbial contamination [29].

Magnetic nanomaterials of the inorganic variety have garnered considerable attention in the research community owing to their unique magnetic properties [48,49]. These materials not only possess the inherent advantages associated with inorganic materials but also offer the distinct advantage of easy separation through the application of magnetic fields, thereby enhancing their reusability [25,50]. It is worth noting that magnetic nanomaterials can be further enhanced through surface modification, which serves to improve the stability and repeatability of PGA immobilization by means of covalent crosslinking [51,52]. Furthermore, covalent crosslinking stands as a solution to address the challenges related to the propensity for easy detachment in physical adsorption techniques [53–55], as well as the issues of low catalytic efficiency that can arise from embedding methods [56]. It is worth mentioning that ferrite is widely used in environmental, biological and other fields. For example, Hublikar et al. demonstrated the adsorption capacity of Zn-Co ferrite nanoparticles and their efficiency in removing contaminants from environmental dyes, and described the mechanism of their interaction with dyes [57]. Kazemi et al. successfully synthesized bismuth and cobalt doped ferrite for tramadol degradation in water samples [58]. Nazir et al. synthesized lanthanum ferrites for the degradation of cefadroxil [59], all of which showed strong photocatalytic properties. Dhanda et al. prepared nickel-cobalt ferrite and found that it has a significant inhibitory effect on the human fungal pathogen Candida albicans [60]. Not only these, ferrite is also emerging in cancer treatment. For example, Ahmad et al. found that manganese cobalt ferrite had obvious inhibitory effect on human breast cancer cells [61]; Wang et al. modified manganese-zinc ferrite with polyethylene glycol and showed great synergistic anti-tumor effects in vivo [62].

In this study, we explored the application of ferrite in enzyme immobilization processes. Specifically, we focused on the use of magnetic Ni_0.4_Cu_0.5_Zn_0.1_Fe_2_O_4_ nanoparticles, which were prepared using an advanced, convenient, simple, and rapid combustion process. To enhance the nanoparticles’ functionality, a SiO_2_ functional layer was applied to their surface using sodium silicate [63,64]. The modification process with sodium silicate introduced a substantial amount of Si-OH groups on the surface of the nanoparticles. Subsequently, we developed the immobilized PGA (magnetic Ni_0.4_Cu_0.5_Zn_0.1_Fe_2_O_4_-SiO_2_-GA-PGA nanoparticles) by crosslinking the modified magnetic Ni_0.4_Cu_0.5_Zn_0.1_Fe_2_O_4_-SiO_2_ nanoparticles with the potent covalent crosslinking agent, glutaraldehyde [65–67]. This method of immobilization ensured a stable and strong bonding between the PGA enzyme and the magnetic nanoparticles, enhancing the overall performance of the system. In-depth studies were conducted to determine the optimum preparation conditions, catalytic conditions, and enzymatic properties of the magnetic Ni_0.4_Cu_0.5_Zn_0.1_Fe_2_O_4_-SiO_2_-GA-PGA nanoparticles. The results demonstrated that these nanoparticles exhibited significantly higher stability and repeatability, making them promising candidates for enzyme immobilization applications. This research represented a significant step forward in the development of efficient and stable enzyme immobilization techniques, with potential applications in various fields.

## Experimental

### Materials and instruments

#### Materials

Penicillin G acylase (90%, CAS: 9014-06-6, Zhejiang Wild Wind Pharmaceutical Co., Ltd). Penicillin G potassium salt (98.0%, CAS: 113-98-4, Shanghai Maclin Biochemical Technology Co., LTD), P-dimethylaminobenzaldehyde (PDAB) (98.0%, CAS: 100-10-7, Shanghai Aladdin Reagent Co., Ltd). Coomassie Brilliant Blue G250 (AR, CAS: 6104-58-1, Beyotime Biotechnology). Sodium hydroxide (AR, CAS: 1310-73-2), Glacial acetic acid (99.99%, CAS: 64-19-7), Sodium dihydrogen phosphate dihydrate (99.0%, CAS: 13472-35-0), 25% glutaraldehyde solution (BR, CAS: 111-30-8), Hydrochloric acid (AR, CAS: 7647-01-0), 6-aminopicillanic acid (6-APA) (98.0%, CAS: 551-16-6), Sodium silicate 9-hydrate (AR, CAS: 13517-24-3), Nickel nitrate hexahydrate (98.0%, CAS: 13478-00-7), Copper nitrate trihydrate (99.0%, CAS: 10031-43-3), Zinc nitrate trihydrate (98.0%, CAS: 10196-18-6), Iron nitrate hexahydrate (98.5%, CAS: 7782-61-8) (Sinopharm Chemical Reagent Co., Ltd). Sodium chloride (99.8%, CAS: 7647-14-5), Phosphoric acid (HPLC, CAS: 7664-38-2) (Chengdu Chron Chemicals Co., Ltd). Anhydrous disodium hydrogen phosphate (99.99%, CAS: 7558-79-4, Shanghai Zhanyun Chemical Co., Ltd). Anhydrous ethanol (AR, 99.7%, CAS: 64-17-5, Xilong Scientific Co., Ltd). Phosphate buffered saline (1M, Servicebio Biological, Wuhan).

#### Instruments

Water bath thermostatic oscillator (SHZ-88), Digital display thermostatic water bath (HH-S_2_) (Jiangsu Jintan Medical Instrument Factory). Precision acidity meter (PHS-3C) (Shanghai Shengke Instrument Co., Ltd). Vacuum drying oven (DZF-6020) (Shanghai Yiheng Technology Co., Ltd). Analytical balance (FA1004) (Shanghai Anting Electronic Instrument Factory). Thermo Fisher centrifuge (Thermo Fisher centrifuge) (Thermo Fisher Scientific Inc.). Ultraviolet-visible spectrophotometer (UV-2450/2550) (Shimadzu Production House, Japan). Programmed temperature control box type high-temperature sintering furnace (KSL-1200X) (Hefei Kejing Material Technology Co., Ltd). Rigaku D/max 2500 PC X-ray diffraction (Bruker, D8 ADVANCE, Germany) with Cu-Kα radiation at the conditions: 2 theta from 20°- 80° range, the step was 0.01918°, the counting time/step was 0.8609, and the scanning rate was 7°·min^-1^. The ADE DMS-HF-4 vibrating sample magnetometer (VSM) with the test magnetic field range: -15 kOe to +15 kOe at room temperature. Transmission Electron Microscope (Hitachi HT7800, Japan. Power Supplyoutput Rating: AC100 V 10 A, DC65 V 17.5 A). High Resolution Transmission Electron Microscopy (TF20, Acceleration voltage: 200 kV, Magnification, 25 K-1030 K, Point resolution, 0.24 nm, Line resolution: 0.102 nm, Information resolution, 014 nm, Sample dumping Angle: <±30). And Fully Automatic Specific Surface and Aperture Distribution Analyzer (Quantachrome, NOVA 3000e, America. Porosity 0.35–500 nm, Specific surface area 0.01 m^2^·g^-1^).

### Preparation of magnetic Ni_0.4_Cu_0.5_Zn_0.1_Fe_2_O_4_ nanoparticles

**Fig 1:**
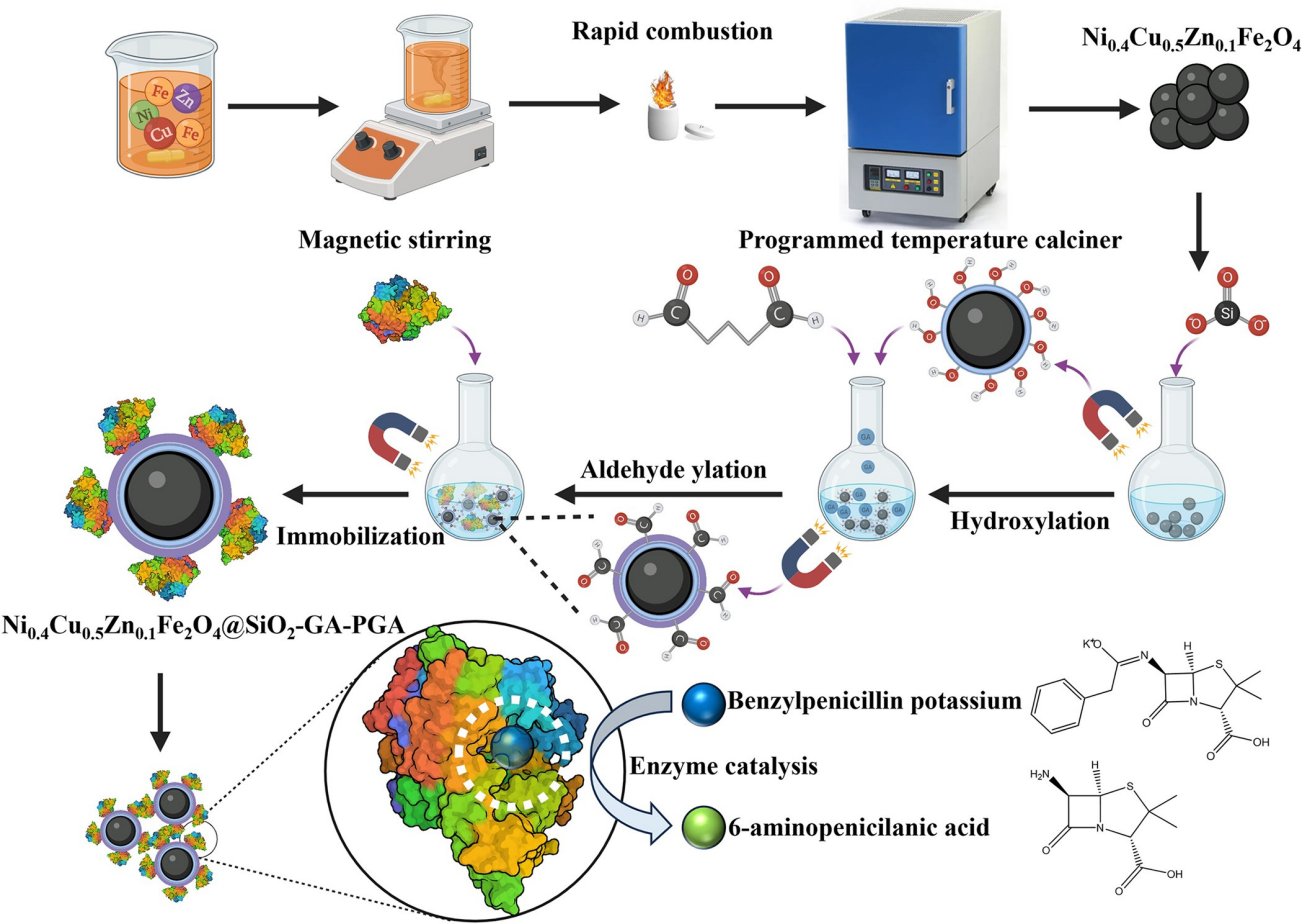
The material preparation and immobilization process.

The preparation process of the magnetic Ni_0.4_Cu_0.5_Zn_0.1_Fe_2_O_4_ nanoparticles was shown in Fig 1. In a beaker containing 20 milliliters of anhydrous ethanol, 0.87 g of nickel nitrate hexahydrate, 1.08 g of copper nitrate trihydrate, 0.25 g of zinc nitrate hexahydrate, and 6.91 g of ferric nitrate nonahydrate were combined according to the molar ratio of Ni, Cu, Zn, and Fe as 4:5:1:20. The content should become a homogeneous and clear solution through 30 min of magnetic stirring. The homogeneous solution was transferred into a crucible, followed by ignition using a lighter, allowing it to burn until self-extinguished. It was worth mentioning that the crucible should be placed in a non-sealed area that permitted airflow. These locations may be either in a laboratory, indoors or outdoors, with proper protective measures or safety protocols in place. When the temperature of the crucible was consistent with room temperature, the crucible was taken into a programmed temperature control box type high-temperature sintering furnace and calcined at 400°C for 2.0 h with a heating rate of 3°C·min^-1^. The magnetic Ni_0.4_Cu_0.5_Zn_0.1_Fe_2_O_4_ nanoparticles which looked dark brown had been prepared.

### Modification of magnetic Ni_0.4_Cu_0.5_Zn_0.1_Fe_2_O_4_ nanoparticles

The 1.0 g magnetic Ni_0.4_Cu_0.5_Zn_0.1_Fe_2_O_4_ nanoparticles were evenly dispersed at 200 mL double distilled water. When the suspension water bath was heated to 80°C, the 10 mL 1 M sodium silicate was added into the suspension while stirring vigorously. Then, the 2 M HCl was used to keep the pH of the reaction system at 6. The reaction was stirred continuously at 80°C for 30 min. The magnetic Ni_0.4_Cu_0.5_Zn_0.1_Fe_2_O_4_@SiO_2_ nanoparticles had been obtained as shown in Fig 1.

The magnetic Ni_0.4_Cu_0.5_Zn_0.1_Fe_2_O_4_@SiO_2_ nanoparticles were prepared into 0.1g/mL suspension with PBS (phosphate buffered saline) (0.1 M, pH = 7.2). An appropriate amount of nanoparticles suspension was added with glutaraldehyde (25%) according to the volume ratio of 5:1 and kept stirring for 2 h. After the reaction, it was washed with normal saline several times and the magnetic Ni_0.4_Cu_0.5_Zn_0.1_Fe_2_O_4_@SiO_2_-GA nanoparticles had been gained.

### Immobilization of penicillin G acylase

The PGA solution was diluted 45 times with PBS (pH = 8). The process of immobilization of PGA was shown in Fig 1. The 0.1 g magnetic Ni_0.4_Cu_0.5_Zn_0.1_Fe_2_O_4_@SiO_2_-GA nanoparticles were put into 5 mL PGA diluent, and shaken for a while with 25°C, and 115 r·min^-1^ in the water bath thermostatic oscillator. After the reaction, the immobilized PGA was obtained by centrifugation and washing precipitation with PBS. It was also important to recover the supernatant and washing liquid and subsequently added the Coomassie Brilliant Blue (CBB-G250) for measurement of the free PGA concentration. Because CBB-250 could bind with protein not only to produce a color reaction but also to produce strong ultraviolet absorption, it was used to determine the concentration of PGA protein.

### Standard curve establishment and Optimum condition study

Bovine serum albumin was prepared as a 100 μg·mL^-1^ working solution using an appropriate amount of double-distilled water. This solution was further diluted with 0.9% NaCl to generate a series of gradient concentrations, maintaining an equal concentration gradient difference. Subsequently, 5 mL of CBB-G250 was added to each tube, and the mixture was allowed to sit for 5 minutes. The protein concentration was determined by measuring the absorbance at a wavelength of 595 nm using an Ultraviolet-visible spectrophotometer. For the 6-APA, a 5 mM solution was prepared by weighing and dissolving it, with the addition of a small amount of hydrochloric acid to aid in dissolution. The 6-APA solution was then diluted at equal intervals to create a gradient dilution ranging from 0 to 4 mM concentration. In each tube, 3.5 mL of 5 mM PDAB was added, and the mixture was allowed to stand for 5 minutes. The concentration of 6-APA was determined by measuring the absorbance at a wavelength of 415 nm using an Ultraviolet-visible spectrophotometer. This protocol allowed accurate analysis of bovine serum albumin and 6-APA concentrations, providing a comprehensive linear calculation range for subsequent studies.

The 0.1 g magnetic Ni_0.4_Cu_0.5_Zn_0.1_Fe_2_O_4_@SiO_2_-GA nanoparticles were uniformed dispersion into a 4.6 mL PGA solution. To study the optimal immobilization time, the shaking was carried out at 115 r·min^-1^ for 6, 12, 18, and 24 h. Similarly, 0.05, 0.10, 0.15, and 0.2 mL of PGA mother liquor were taken, diluted to 4.6 mL with PBS (pH = 8), the 0.1g magnetic Ni_0.4_Cu_0.5_Zn_0.1_Fe_2_O_4_@SiO_2_-GA nanoparticles were added, and the effect of PGA concentration on the immobilization process was studied after 115 r·min^-1^ and shaking for 18 h.

### Catalytic condition, Kinetic study, and Reuse performance of free PGA and immobilized PGA

The 4 μL free PGA and 0.1 g immobilized PGA were uniformly dispersed in PBS of different pH (6–9). After being kept at room temperature for 5 min, 5 mL of 4% penicillin K solution at the same temperature was added to each tube for 10 min. 1 mL supernatant was diluted five times to determine the activity of free enzymes.

The optimal pH for catalysis was selected, and 2 mL of free PGA and immobilized PGA (suspended with PBS) were insulated at different temperatures (20–60°C) for 5 minutes, respectively, and then the enzyme activity was measured by the same method. The free and immobilized PGA were kept at different temperatures (30, 40, 50, and 60°C) for different times (2, 4, 6, 8, and 10 h). The influence of time on the catalytic performance of PGA was studied.

### Michaelis constant

The initial hydrolysis rates of immobilized and free PGA were determined at 20°C with different concentrations of penicillin K solution (0.05 mol·L^-1^, 0.025 mol·L^-1^, 0.017 mol·L^-1^, 0.0125 mol·L^-1^, 0.01 mol·L^-1^) as substrate. The enzymatic properties of immobilized PGA were studied by the Lineweaver-Burk diagram with the reciprocal concentration of substrate (L·mol^-1^) as the horizontal coordinate and the reciprocal initial reaction velocity (min·μmol^-1^) as the vertical coordinate.

The 0.1 g immobilized PGA was added into 2 mL PBS (pH = 8). The suspension was allowed to stand at room temperature for 5 minutes and then 5 mL penicillin K solution was added to react for 10 minutes. After separation with magnets, cleaning and drying continued the above experiment. The catalytic activity of each PGA was measured separately.

## Results and discussion

### Characterization of magnetic Ni_0.4_Cu_0.5_Zn_0.1_Fe_2_O_4_ nanoparticles

**Fig 2:**
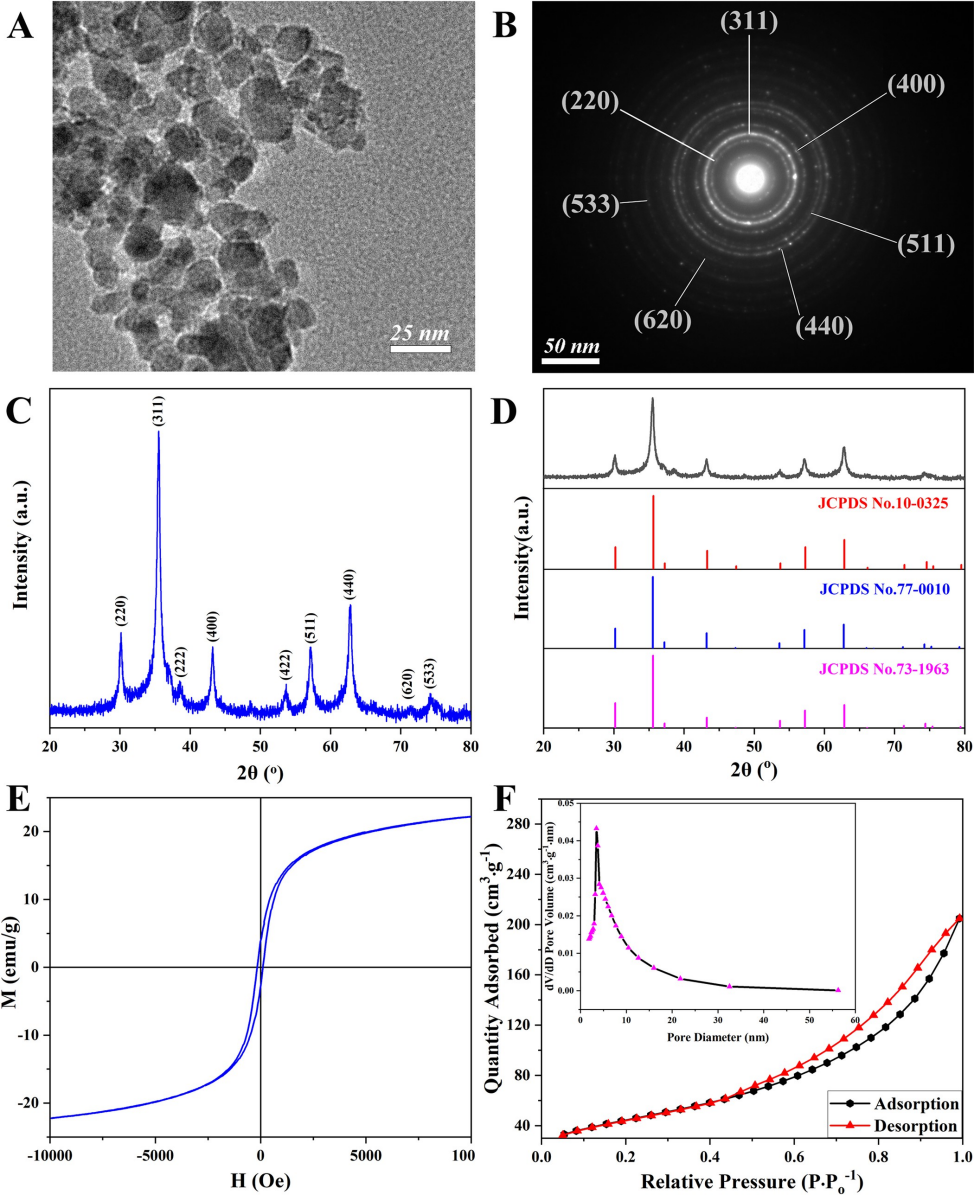
Transmission electron microscopy (TEM) image (A), selected area electron diffraction (SAED) spectrum (B), X-ray diffraction (XRD) pattern (C), XRD standard card (D), vibrating sample magnetometry (VSM) curve (E), and Nitrogen adsorption isotherm and pore distribution map (F) of the magnetic Ni_0.4_Cu_0.5_Zn_0.1_Fe_2_O_4_ nanoparticles prepared at 400°C for 2.0 h with a heating rate of 3°C·min^-1^.

**Fig 3:**
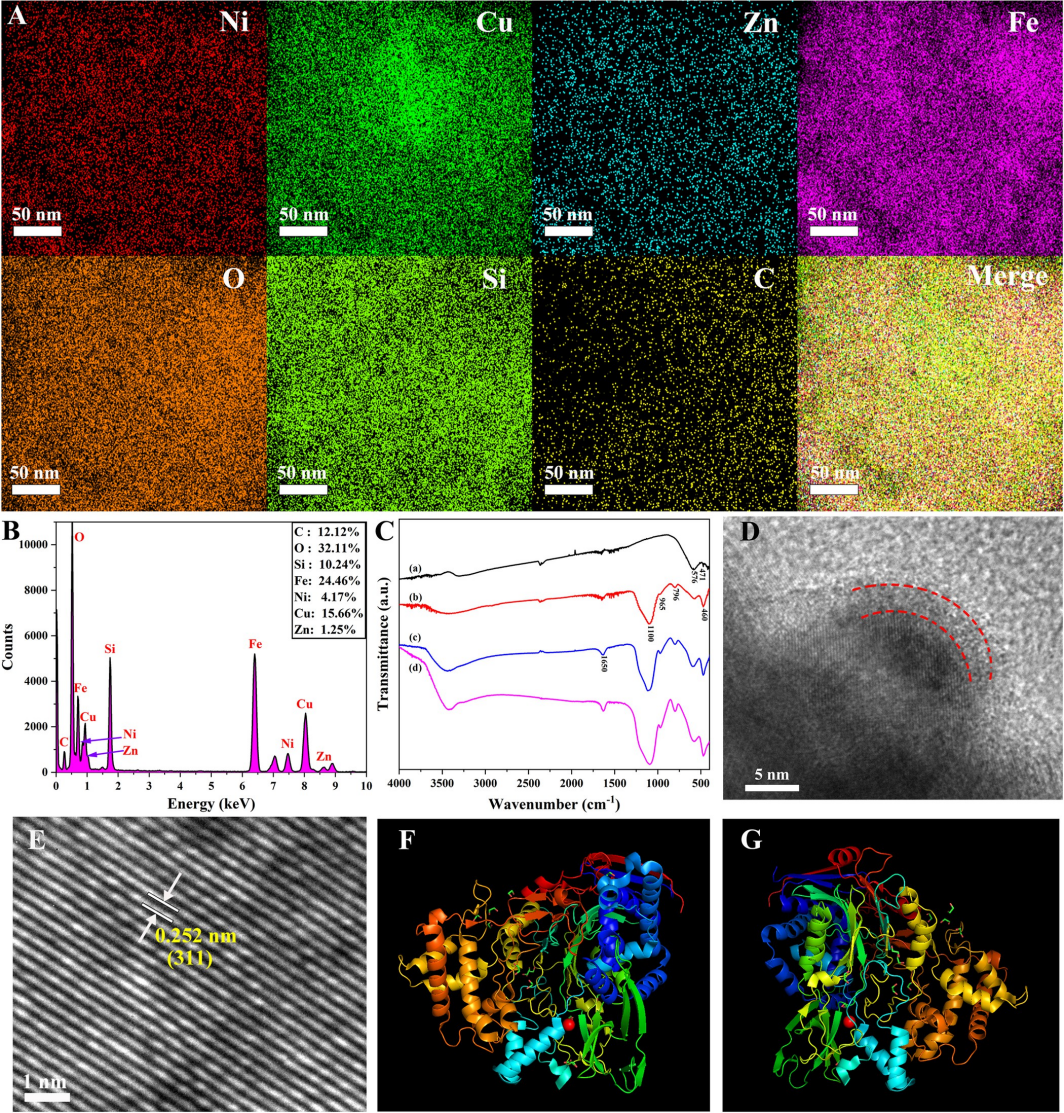
The EDS maps (A) and EDS spectrogram (B) of Ni_0.4_Cu_0.5_Zn_0.1_Fe_2_O_4_@SiO_2_-GA, FTIR spectra (C) of Ni_0.4_Cu_0.5_Zn_0.1_Fe_2_O_4_ nanoparticles (a), Ni_0.4_Cu_0.5_Zn_0.1_Fe_2_O_4_@SiO_2_ (b), Ni_0.4_Cu_0.5_Zn_0.1_Fe_2_O_4_@SiO_2_-GA (c) and Ni_0.4_Cu_0.5_Zn_0.1_Fe_2_O_4_@SiO_2_-GA-PGA (d), the HRTEM gram (D) and Lattice fringe picture (E) of Ni_0.4_Cu_0.5_Zn_0.1_Fe_2_O_4_@SiO_2_-GA, the docking pattern of penicillin G and PGA (F), and the molecular model of PGA (G).

The TEM image of the magnetic Ni_0.4_Cu_0.5_Zn_0.1_Fe_2_O_4_ nanoparticles (Fig 2A) clearly showed spheroidal or spheroidal polycrystalline particles with uniform particle size distribution, with an average particle size of 11.7 nm. The SAED (Selected Area Electron Diffraction) pattern in Fig 2B confirmed that the magnetic nanoparticles of Ni_0.4_Cu_0.5_Zn_0.1_Fe_2_O_4_ were polycrystalline materials with distinct crystal planes. The XRD pattern further confirmed that the Ni_0.4_Cu_0.5_Zn_0.1_Fe_2_O_4_ nanoparticles possessed a spinel crystal structure. The theoretical grain size was determined to be 11.0411 nm, as illustrated in Fig 2C. The characteristic peaks of 30.16°, 35.52°, 38.50°, 43.2°, 53.68°, 57.12°, 62.88°, 71.00°, and 74.28° were consistent with the results of SAED, corresponding to (220), (311), (222), (400), (422), (511), (440), (620), and (533) crystal plane diffraction. Just as illustrated in Fig 2D, the XRD pattern was compared to the standard card in of NiFe_2_O_4_ (JCPDS No.10-0325), CuFe_2_O_4_ (JCPDS No.77-0010), and ZnFe_2_O_4_ (JCPDS No.73-1963), and the main characteristic peaks of the standard card could correspond to the characteristic peaks of the magnetic Ni_0.4_Cu_0.5_Zn_0.1_Fe_2_O_4_ nanoparticles. Additionally, Fig 3D and 3E were high-magnification HRTEM images of localized regions of Ni_0.4_Cu_0.5_Zn_0.1_Fe_2_O_4_ nanoparticles. As depicted in Fig 3E, the sample exhibited distinct lattice stripes that were well-organized with no evident defect structure, signifying a high degree of crystallization in the nanoparticles. The calculated crystal surface spacing was 0.252 nm, corresponding to the characteristic Ni_0.4_Cu_0.5_Zn_0.1_Fe_2_O_4_ cubic spinel structure (311). This observation suggested that the Ni_0.4_Cu_0.5_Zn_0.1_Fe_2_O_4_ nanoparticles preferentially grew along the (311) crystal surface, aligning with the previously discussed XRD results. Subsequently, Fig 2E showed that the VSM of the magnetic Ni_0.4_Cu_0.5_Zn_0.1_Fe_2_O_4_ nanoparticles, the saturation magnetization, remanent magnetism, and coercivity were respectively 22.6 emu·g^-1^, 2.8 emu·g^-1^, and 2.8 Oe, indicating that it was a soft magnetic material with superior magnetic properties, and that could be used in the field of enzyme immobilization to improve enzyme recovery and save costs. In the BET report (Fig 2F), the surface area was 159.9289 m^2^·g^-1^, the sample density was 1.0 g·cm^-3^, the particle size (DBET) that could be calculated according to the formula was 37.5167 nm, and the reason for such a large size might be that the prepared nanoparticles were not regular shaped and regular spherical particles. The pore volume (Vp) was 0.313416 cm^3^·g^-1^, the Pore radius (Dv) was 1.74101 nm, and the pore diameter (Dp) was 3.48021 nm.

### Immobilization characterization of penicillin G acylase and Molecular docking studies

To confirm the successful modification of nanoparticles, EDS technology was employed for characterization. Fig 3A presented EDS maps illustrating various elements and their superposition and Fig 3B illustrated the element content in detail. It was preliminarily proved that the nanoparticles were coated. Fig 3C showed the FTIR spectra of Ni_0.4_Cu_0.5_Zn_0.1_Fe_2_O_4_ nanoparticles (a), Ni_0.4_Cu_0.5_Zn_0.1_Fe_2_O_4_@SiO_2_ (b), Ni_0.4_Cu_0.5_Zn_0.1_Fe_2_O_4_@SiO_2_-GA (c) and Ni_0.4_Cu_0.5_Zn_0.1_Fe_2_O_4_@SiO_2_-GA-PGA (d). The peaks at 471 cm^-1^ and 576 cm^-1^ were the stretching vibrations of the bond between metal and oxygen elements [68]. The strong and wide band of 1100 cm^-1^ was attributed to Si-O-Si antisymmetric stretching vibration. The peaks at 796 cm^-1^ could be considered to be Si-O symmetric stretching vibration and the peak at about 460 cm^-1^ could be considered to be Si-O bond stretching vibration, and the peak at about 965 cm^-1^ was ascribed to Si-OH stretching vibration, which indicated that Ni_0.4_Cu_0.5_Zn_0.1_Fe_2_O_4_@SiO_2_ was successfully prepared and the presence of Si-OH in the outer layer of nanoparticles was beneficial to surface modification. The wide peak at 3340 cm^-1^ was the antisymmetric stretching vibration peak of the O-H of the structure water. Compared with curve (c), the peak signal strength of 1100 cm^-1^ was enhanced in curve (d), and it might be the N-H vibration of imino after PGA binding. In summary, it could be concluded that the nanoparticles were coated with silica and glutaraldehyde. In addition, to further verify the coating in a more specific and clear manner, high-resolution transmission electron microscopy was used to characterize its morphology (Fig 3D and 3E). The coating around the nanoparticles could be seen (between the red lines).

The docking pattern of penicillin G and PGA was exhibited in Fig 3F. The center of enzyme activity of PGA was like a square pocket. Many active sites that played an important role in catalysis were found at the putative site (PDB ID: 1GM8) where potassium penicillin bound to PGA, and the substrate molecules were very close to PGA residues due to steric hindrance, so they could show high catalytic performance. The optimal molecular binding mode was confirmed by molecular docking, which had great value in studying the conditions of immobilized PGA. Such as glutaraldehyde (GA), a powerful crosslinking [69], bound to free amino acid residues and did not impact PGA activity after cross-linking. In addition, from the molecular model of PGA (Fig 3G), it could be understood that the key groups cross-linked with GA were distributed outside the active center, which further verified that GA cross-linking did not directly affect the catalytic activity and efficiency of PGA [70,71].

### Standard curve, and optimization of the immobilized PGA

**Fig 4:**
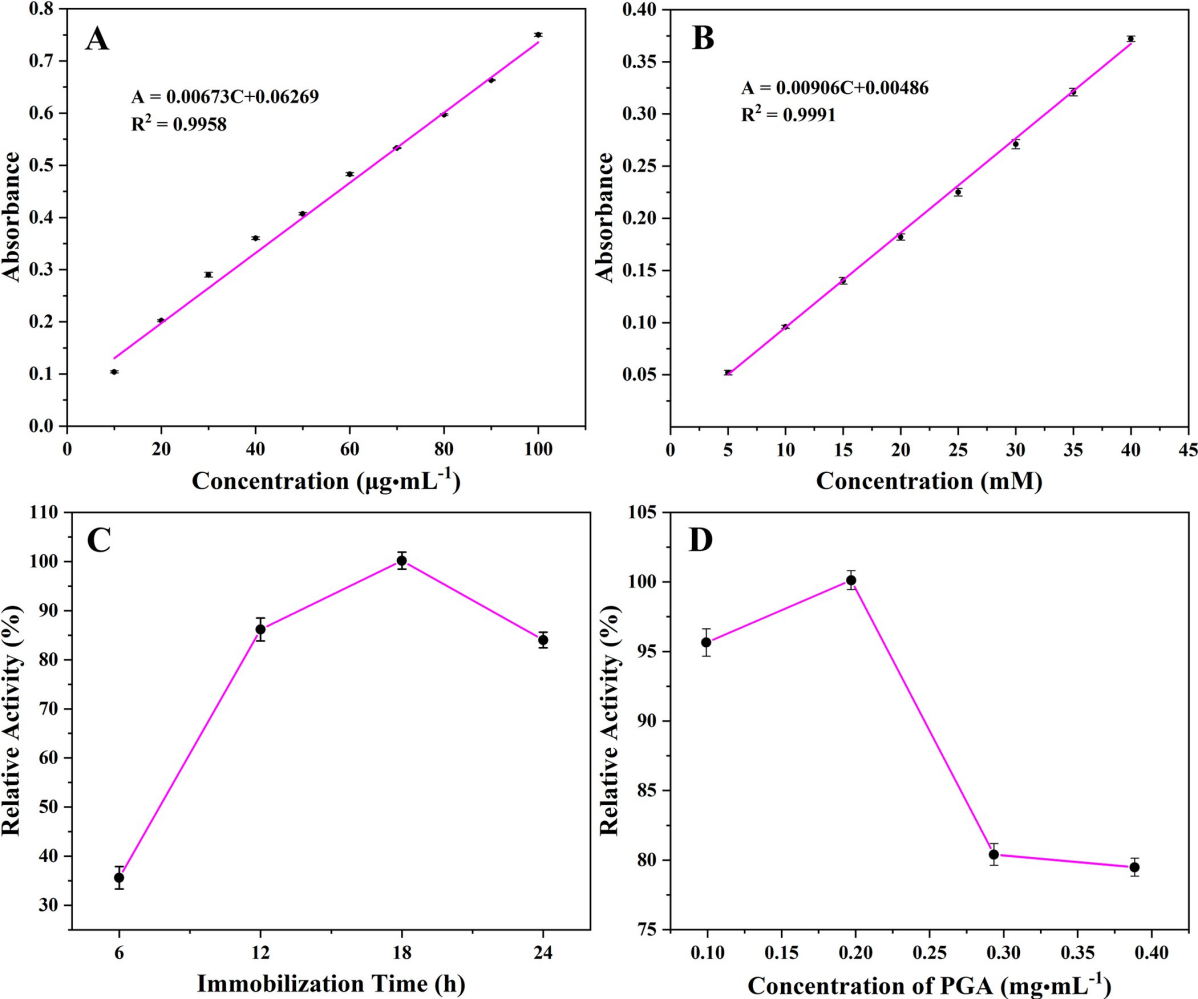
The standard curve of protein (A) and 6-APA (B), the effects of immobilization time (C) and PGA concentration (D) on the catalytic activity of the immobilized enzyme.

Fig 4 showed the standard curve of protein (A) and 6-APA (B). The correlation coefficient of the two lines was greater than 0.99, indicating that they had a good linear relationship and sufficient reliability. The optimization of immobilization time and PGA concentration during immobilization was shown in Fig 4C and 4D, both of which were compared by relative activity. With the extension of immobilization time, the catalytic activity of the immobilized PGA increased gradually, because the cross-linking site of glutaraldehyde and PGA was not saturated, and the cross-linked PGA on the surface of the Ni_0.4_Cu_0.5_Zn_0.1_Fe_2_O_4_ nanoparticles gradually increased, resulting in higher catalytic activity. Unfortunately, when the immobilization time exceeded 18 h, the catalytic activity of immobilized PGA began to decline, which might be because, at 18 h, the binding site of glutaraldehyde and PGA reached saturation, and continued reaction caused excess PGA to enter the surface coating layer of the Ni_0.4_Cu_0.5_Zn_0.1_Fe_2_O_4_ nanoparticles and stay in the gap of immobilized PGA, resulting in the covering of the active site of immobilized PGA. The substrate cannot successfully enter the catalytic center, resulting in greatly reduced catalytic activity [72]. There was another reason for the decrease in activity. With the extension of time, the free PGA in the solution lost its activity, resulting in a decrease in PGA activity after fixation [68]. It could be observed that the increase in PGA concentration caused PGA to compete for binding sites, and when a certain concentration was reached, a competitive inhibition effect was displayed, and a large amount of PGA accumulated and adsorbed on the surface of Ni_0.4_Cu_0.5_Zn_0.1_Fe_2_O_4_ nanoparticles, resulting in the decrease of PGA activity from Fig 4D.

### Stability of the free PGA and immobilized PGA

**Fig 5:**
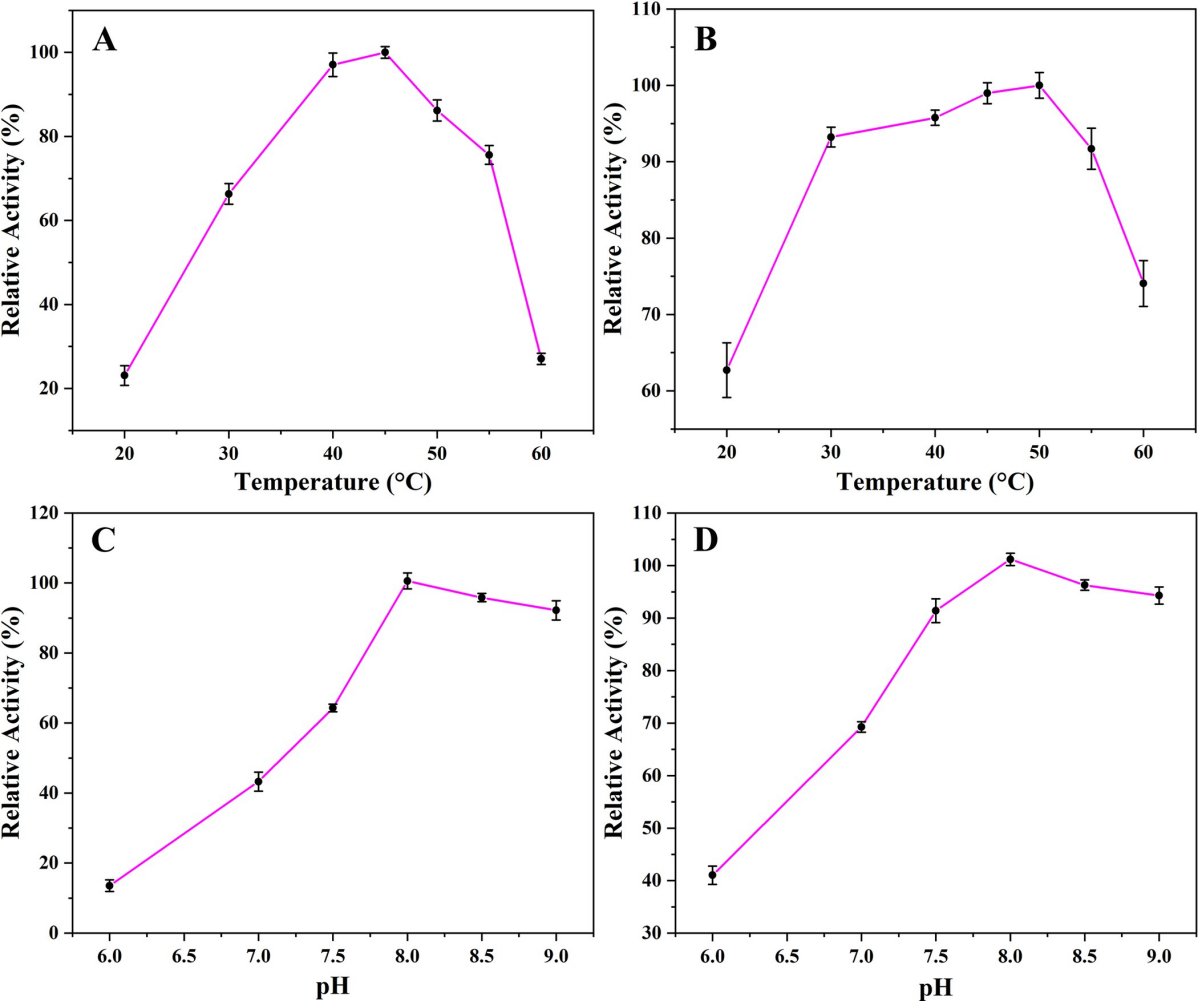
The catalytic capacity of free PGA (A) and immobilized PGA (B) at different temperatures, the effects of different pH on the activity of free PGA (C) and immobilized PGA (D).

Fig 5 showed the relative catalytic properties of free and immobilized PGA over a range of temperatures and pH. The catalytic activity of free PGA was less than 30% at both low and high temperatures, while the minimum catalytic activity of immobilized PGA was more than 65%. This was because after PGA was immobilized, under the influence of the carrier at the same temperature, the advanced structure of PGA was less affected by the temperature, resulting in the catalytic performance of immobilized PGA at extreme temperatures was stronger than that of free PGA and this would be more conducive to PGA storage. The optimal catalytic temperature of immobilized PGA was 50°C, which was 5°C higher than that of free PGA. The catalytic performance of free PGA was greater than 65% in the range of 30–50°C, while the immobilized PGA showed stronger resistance to temperature interference. In this temperature range, the catalytic performance of immobilized PGA was greater than 90%. The excellent catalytic stability of immobilized PGA could show stronger application value in industrial applications (Fig 5A and 5B). pH value could change the conformation of the PGA catalytic site through electrostatic interaction and hydrogen bonding. In addition, the action of strong acids or bases would lead to the degeneration of PGA, which could not respond well to the catalysis of penicillin G potassium salt, and could not play a catalytic role. The optimal catalytic pH of free and immobilized PGA was 8.0. Compared with free PGA, the mechanical properties of nanoparticles improved the mechanical strength of PGA, so it showed strong catalytic performance at different pH and stability of pH change in the catalytic environment (Fig 5C and 5D).

### Thermal stability for free and immobilized PGA

**Fig 6:**
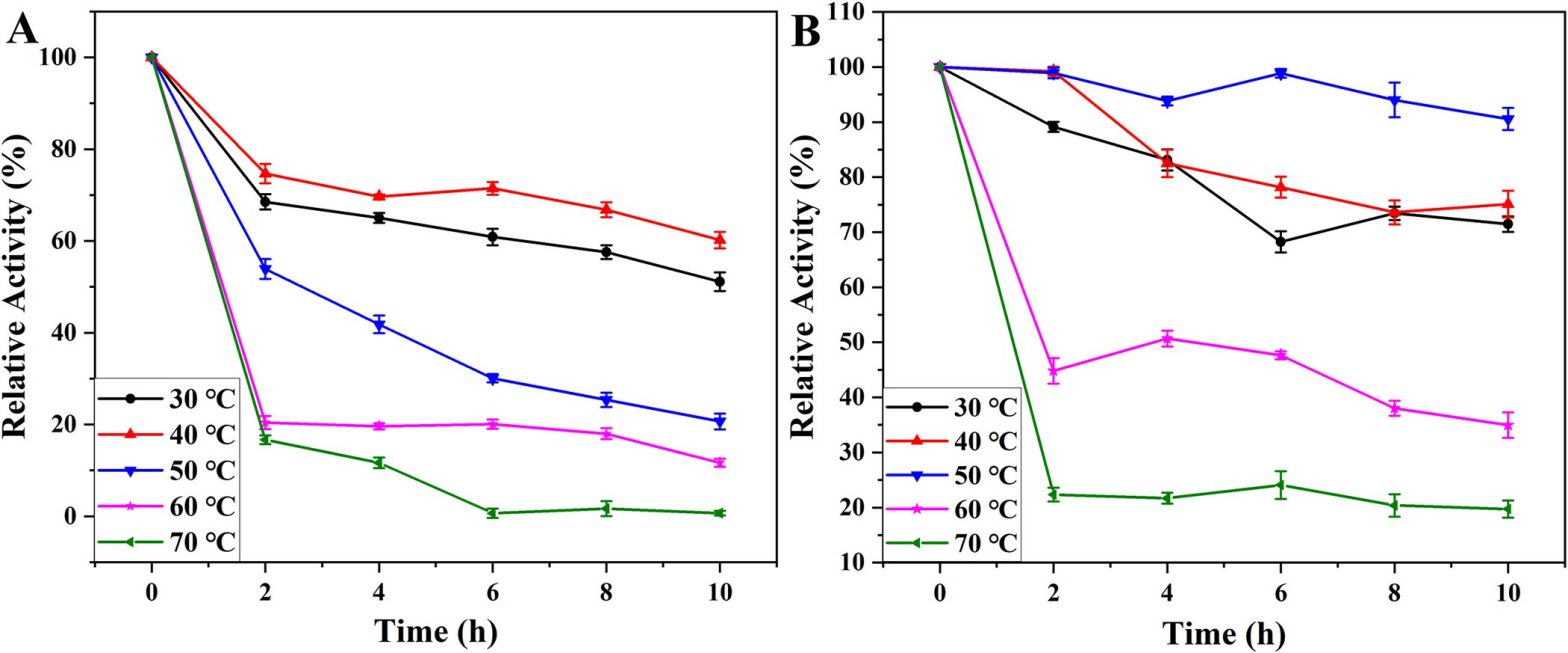
Thermal stability of free PGA (A) and immobilized PGA (B).

The thermal stability of free and immobilized PGA at different incubation times (2, 4, 6, 8, and 10 h) at different temperatures were compared, as shown in Fig 6. No matter at which temperature, with the increase of time, the catalytic performance of PGA was gradually decreased, but the immobilized PGA showed better stability. At low temperatures, immobilized PGA was easier to store than free PGA. At 60°C, the catalytic activity of both decreased dramatically, because when PGA was placed at a higher temperature, the conformational change of aromatic residues in the hydrophobic bag was more intense, so PGA could not respond well to the entry of substrates and play a catalytic role. At 50°C, the immobilized PGA had the strongest catalytic performance and the highest catalytic stability, this could be attributed to the fixation of PGA to the nanoparticles via covalent bonding, thus confining the enzyme to the microstructure, significantly reducing the risk of damage to the protein’s tertiary structure due to increased temperature. This immobilization method greatly enhanced the temperature stability of PGA. In addition, the increase in temperature would enhance the molecular movement, which would increase the contact frequency of the substrate and the enzyme, further improving the catalytic activity [73,74]. To explore the maximum tolerance limit of PGA, higher temperatures were investigated. At 70°C, the catalytic activity of free PGA decreased sharply, reaching about 1.5% in 6 hours. The catalytic activity of immobilized PGA also decreased to about 19%. In summary, at higher temperatures, the increased relative energy heightened molecular motion, causing molecules to deviate from their equilibrium positions and inducing disorder. As a result, the internal structure of protein molecules was disrupted, transforming the original ordered and tightly coiled structure into a disordered, loose, and extended configuration. This led to the exposure of a significant number of hydrophobic groups to the molecular surface, accompanied by a reduction in the distribution of hydrophilic groups on the surface. Consequently, the protein particles became insoluble in water, lost the water film, and the active catalytic pockets were correspondingly compromised [75]. However, when PGA was immobilized on the surface of the carrier, due to the presence of the carrier, the thermal movement of the molecule did not increase as much as that of free PGA, and the threshold of energy required for the protein structure to change from order to disorder was increased. Therefore, even at extreme temperatures (70°C), free PGA basically had no catalytic activity, while immobilized PGA still had weak catalytic efficiency [76].

### Michaelis constants for free and immobilized PGA and Repetition availability of the immobilized PGA

**Fig 7:**
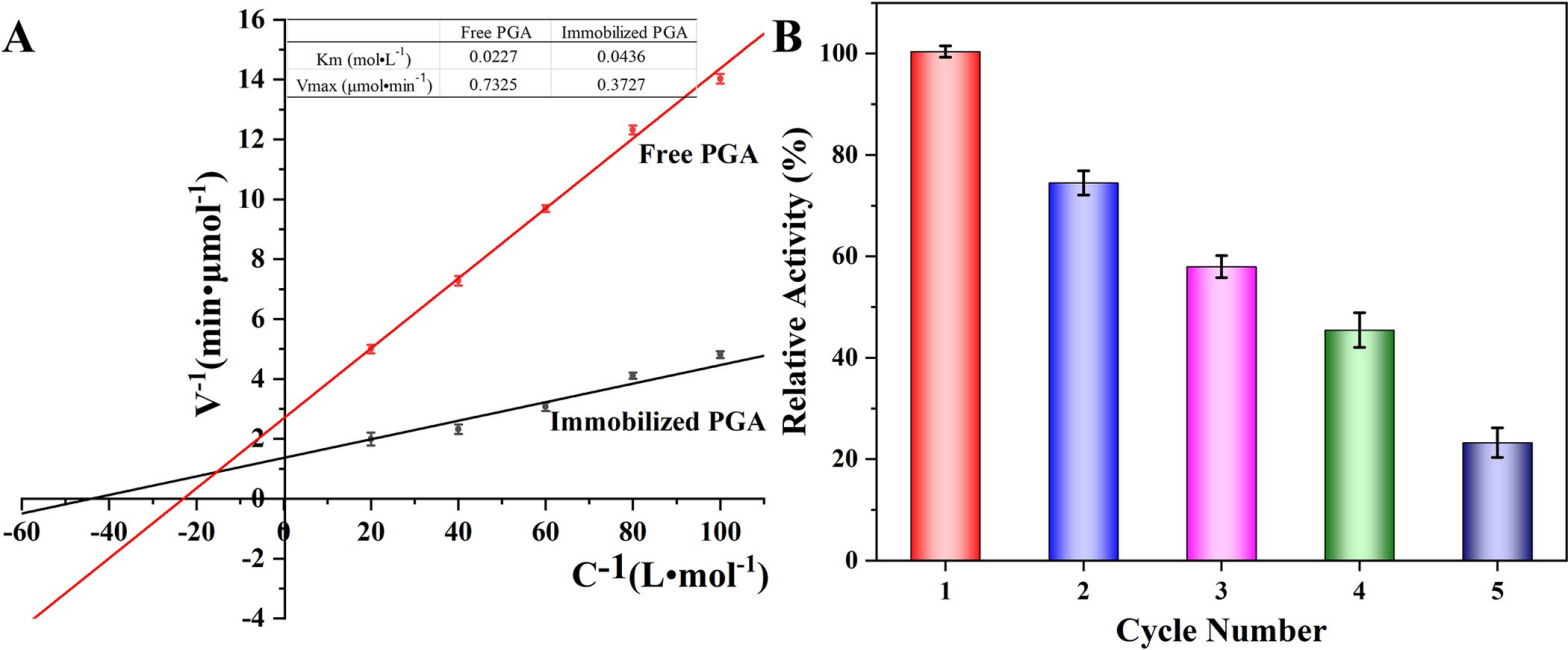
The Michaelis constant of free and immobilized PGA (A), and the Repetitive availability of immobilized PGA (B).

The Michaelis constant equations for both free and immobilized PGA were illustrated in Fig 7A. The V_max_ and K_m_ values for immobilized PGA were determined to be 0.3727 μmol·min^-1^ and 0.0436 mol·L^-1^, respectively, while the corresponding values for free PGA were 0.7325 μmol·min^-1^ and 0.0227 mol·L^-1^. As anticipated, the Km value of immobilized PGA was lower than that of its free counterpart. The immobilization of PGA on the carrier resulted in a localized substrate concentration that was lower. This was attributed to the hindrance caused by steric factors, preventing the substrate from efficiently entering the catalytic pocket. Unlike free PGA, the carrier was unable to uniformly disperse in the solution, leading to a reduction in the catalytic performance of the immobilized PGA. Despite these drawbacks, the advantages of immobilized PGA, such as the ability to be recycled, outweighed these sacrifices. The findings suggest that, in the context of the experiment, the immobilized PGA offered practical benefits that justified the observed limitations.

Subsequently, to assess the reusability of the immobilized PGA, the PGA immobilization underwent separation and washing utilizing a magnetic field, followed by reintroduction into a new catalytic substrate for subsequent reactions. The obtained results revealed that, even after undergoing five cycles, the catalytic efficiency of the immobilized PGA remained above 25%. The decline in catalytic activity was attributed to the partial loss of immobilized PGA during the magnetic separation process and the inevitable denaturation and shedding during successive catalytic reactions. Despite experiencing some losses in catalytic efficiency, the immobilized PGA demonstrated potential cost savings for industrial production. This was attributed to the facile separation of immobilized PGA and the catalyst’s inherent reusability. The ease of recovery and reuse of the immobilized catalyst could contribute to increased efficiency and reduced expenses in large-scale industrial applications.

## Conclusions

In this study, magnetic Ni_0.4_Cu_0.5_Zn_0.1_Fe_2_O_4_ nanoparticles were successfully synthesized via a rapid combustion method. Subsequently, these magnetic Ni_0.4_Cu_0.5_Zn_0.1_Fe_2_O_4_ nanoparticles were surface-modified with sodium silicate and glutaraldehyde for the immobilization of PGA. The enzymatic properties of PGA were investigated before and after immobilization. The immobilized PGA exhibited several notable advantages over its free counterpart. It not only raised the optimal reaction temperature but also maintained a higher catalytic activity at lower temperatures, mitigating the temperature sensitivity drawback associated with free enzymes. Similarly, the immobilized PGA demonstrated excellent catalytic stability within a specific pH range. While the immobilized PGA required a higher substrate concentration to achieve the same catalytic efficiency as the free enzyme, it retained over 25% of its catalytic activity after five cycles of catalysis, offering significant cost savings. Overall, this study holds significant importance as it provides valuable insights into the immobilization of enzymes using magnetic inorganic nanoparticles, shedding light on the development of novel methods for maintaining enzyme performance and recyclability.

The rapid combustion method enables expedited synthesis of ferrite, while the incorporation of various transition metal elements offers distinct functionalities within biological systems. For instance, copper and iron have been shown to mediate the apoptosis of cancer cells. In future investigations, doping ferrites with different elements can be explored. Furthermore, through surface modification, insoluble drugs can be loaded onto the ferrite nanoparticles and delivered to the targeted site by an external magnetic field. The small size and magnetic targeting properties of these nanoparticles may also present novel strategies for overcoming the blood-brain barrier (BBB) and demonstrate the potential of using magnetic inorganic nanoparticles in the field of biology.

## Supporting information

S1 Data The raw data reports generated by the measuring instruments during the experiment are presented in the worksheet in support Information 1, which details the raw data under each test target. The original data measured and calculated during the experiment were made into tables named after the experiment content and presented in supporting Information 2. Details of the data designed in all experiments can be found in the supporting documentation.
(XLSX)

S1 File (DOCX)
