# Supplementary material for: Enhanced catalytic performance of penicillin G acylase by covalent immobilization onto functionally-modified magnetic Ni0.4Cu0.5Zn0.1Fe2O4 nanoparticles
Source: PLoS One. 2024 Jan 19;19(1):e0297149. doi: 10.1371/journal.pone.0297149 (PMC10798532; doi:10.1371/journal.pone.0297149)
Supplement: S1 File — (DOCX) [file pone.0297149.s002.docx]

**Supporting Information**

**Enhanced catalytic performance of** **penicillin G acylase by covalent immobilization onto functionally-modified magnetic Ni_0.4_Cu_0.5_Zn_0.1_Fe_2_O_4_ nanoparticles**

Zhixiang Lv^1^, Zhou Wang^2^, Shaobo Wu^3*^ and Xiang Yu^2*^

^1^ The People’s Hospital of Danyang, Affiliated Danyang Hospital of Nantong University, Zhenjiang 212300, P.R. China

^2^ College of Vanadium and Titanium, Panzhihua University, Panzhihua 617000, P.R. China

^3^ Zhenjiang Hospital of Chinese Traditional and Western Medicine, Zhenjiang 212013, P.R. China

*Corresponding author:

E-mail address: zjwushaobo@163.com (Shaobo Wu)

E-mail address: pzhyu_xiang@163.com (Xiang Yu)

All raw data included in the experiment will be presented in this supporting information. The characterization diagram shown in Fig 2 and Fig 3, including XRD, VSM, EDS, FTIR, nitrogen adsorption and desorption isotherm and pore distribution, was not suitable for presentation here because the data table was too long. The detailed data could be queried from the worksheet of the ‘Supporting Information’.

Table S1 Standard curve of protein concentration

| Concentration | Absorbance | | |
| --- | --- | --- | --- |
| (μg·mL^-1^) | 1 | 2 | 3 |
| 10 | 0.104 | 0.1 | 0.102 |
| 20 | 0.202 | 0.203 | 0.205 |
| 30 | 0.29 | 0.299 | 0.294 |
| 40 | 0.36 | 0.363 | 0.365 |
| 50 | 0.407 | 0.41 | 0.411 |
| 60 | 0.483 | 0.489 | 0.486 |
| 70 | 0.533 | 0.531 | 0.532 |
| 80 | 0.597 | 0.595 | 0.594 |
| 90 | 0.663 | 0.661 | 0.661 |
| 100 | 0.75 | 0.756 | 0.752 |

Table S2 Standard curve of 6-APA

| Concentration | Absorbance | | |
| --- | --- | --- | --- |
| (mM) | 1 | 2 | 3 |
| 5 | 0.052 | 0.054 | 0.056 |
| 10 | 0.096 | 0.097 | 0.099 |
| 15 | 0.14 | 0.135 | 0.141 |
| 20 | 0.182 | 0.179 | 0.185 |
| 25 | 0.225 | 0.23 | 0.232 |
| 30 | 0.271 | 0.264 | 0.272 |
| 35 | 0.321 | 0.323 | 0.328 |
| 40 | 0.372 | 0.37 | 0.375 |

Table S3 Probe into the optimal immobilization time of PGA

| Immobilization Time | Relative Activity (%) | | |
| --- | --- | --- | --- |
| (h) | 1 | 2 | 3 |
| 6 | 35.60285 | 33.42451 | 37.98515 |
| 12 | 86.15792 | 83.29417 | 87.91845 |
| 18 | 100 | 98.65651 | 96.58535 |
| 24 | 84.01499 | 82.69545 | 85.86542 |

Table S4 Probe into the optimal immobilization concentration of PGA

| Concentration of PGA | Relative Activity (%) | | |
| --- | --- | --- | --- |
| (mg/mL) | 1 | 2 | 3 |
| 0.09918 | 95.64316 | 95.02393 | 96.95903 |
| 0.19697 | 100 | 99.84505 | 98.74771 |
| 0.29339 | 80.4 | 79.2888 | 80.7951 |
| 0.38847 | 79.48246 | 79.78988 | 78.55025 |

Table S5 Exploration of optimal catalytic temperature of free PGA

| Temperature | Relative Activity (%) | | |
| --- | --- | --- | --- |
| ℃ | 1 | 2 | 3 |
| 20 | 23.08824 | 22.23852 | 26.628 |
| 30 | 66.32353 | 63.08755 | 67.95954 |
| 40 | 97.05882 | 98.9751 | 94.0125 |
| 45 | 100 | 97.52358 | 99.90495 |
| 50 | 86.17647 | 88.4076 | 83.41031 |
| 55 | 75.58824 | 79.81564 | 78.9498 |
| 60 | 27.05882 | 29.03015 | 26.49675 |

Table S6 Exploration of optimal catalytic pH of free PGA

| pH | Relative Activity (%) | | |
| --- | --- | --- | --- |
|  | 1 | 2 | 3 |
| 6.0 | 11.42135 | 10.22126 | 13.51459 |
| 7.0 | 40.3515 | 45.83684 | 43.24714 |
| 7.5 | 62.04104 | 62.94582 | 64.27307 |
| 8.0 | 97.36412 | 97.00511 | 100 |
| 8.5 | 93.9654 | 93.7955 | 95.81932 |
| 9.0 | 89.01563 | 93.0345 | 92.17594 |

Table S7 Exploration of optimal catalytic temperature of immobilization PGA

| Temperature | Relative Activity (%) | | |
| --- | --- | --- | --- |
| ℃ | 1 | 2 | 3 |
| 20 | 59.25975 | 66.40245 | 62.71186 |
| 30 | 92.12534 | 90.914 | 93.22034 |
| 40 | 94.28451 | 96.3124 | 95.76271 |
| 45 | 97.09865 | 96.96183 | 98.98305 |
| 50 | 99.03245 | 96.8851 | 100 |
| 55 | 88.95246 | 87.09654 | 91.69492 |
| 60 | 68.45616 | 69.53512 | 74.0678 |

Table S8 Exploration of optimal catalytic pH of immobilization PGA

| pH | Relative Activity (%) | | |
| --- | --- | --- | --- |
|  | 1 | 2 | 3 |
| 6.0 | 40.26851 | 41.03999 | 43.61516 |
| 7.0 | 67.39657 | 69.25462 | 68.0147 |
| 7.5 | 89.27934 | 91.40922 | 87.057 |
| 8.0 | 98.0174 | 100 | 99.6874 |
| 8.5 | 97.73252 | 96.29209 | 96.0841 |
| 9.0 | 91.8362 | 94.29683 | 92.84103 |

Table S9 Investigation of thermal stability of free PGA

| Activity (%)  Time (h) | Temperature (℃) | | | | |
| --- | --- | --- | --- | --- | --- |
|  | 30 | 40 | 50 | 60 | 70 |
| 0 | 100 | 98.6742 | 100 | 100 | 99.2658 |
|  | 99.12154 | 100 | 98.523 | 99.2352 | 99.68235 |
|  | 99.54518 | 99.03253 | 99.0325 | 99.02512 | 100 |
| 2.0 | 70.72032 | 77.2352 | 53.85624 | 20.39353 | 17.83512 |
|  | 68.50244 | 78.946 | 56.76162 | 22.68925 | 16.2638 |
|  | 67.45183 | 74.68346 | 52.35725 | 22.02354 | 16.03682 |
| 4.0 | 65.01613 | 69.62586 | 41.31523 | 19.60731 | 13.22635 |
|  | 66.541 | 70.0593 | 44.5394 | 18.32347 | 11.06624 |
|  | 64.39325 | 69.14732 | 40.96542 | 18.80352 | 11.36852 |
| 6.0 | 63.54125 | 69.0253 | 30.07792 | 20.08745 | 2.63329 |
|  | 60.86117 | 71.44541 | 31.86234 | 21.45512 | 1.25103 |
|  | 60.06894 | 69.2325 | 31.35415 | 19.53254 | 0.62358 |
| 8.0 | 57.74471 | 66.78354 | 25.3424 | 17.97808 | 2.95114 |
|  | 55.96325 | 64.0352 | 27.7725 | 18.7325 | 0.32515 |
|  | 58.6852 | 63.965 | 25.02652 | 16.42353 | 1.66823 |
| 10.0 | 51.09761 | 60.19064 | 20.65019 | 11.66916 | 1.12862 |
|  | 53.384 | 57.3652 | 17.31125 | 10.3562 | 0.19835 |
|  | 49.3935 | 56.9523 | 18.43658 | 11.98625 | 0.62257 |

Table S10 Investigation of thermal stability of immobilization PGA

| Activity (%)  Time (h) | Temperature (℃) | | | | |
| --- | --- | --- | --- | --- | --- |
|  | 30 | 40 | 50 | 60 | 70 |
| 0 | 98.68342 | 99.32515 | 99.75135 | 99.15125 | 98.21525 |
|  | 100 | 98.2364 | 99.03581 | 98.51534 | 100 |
|  | 99.3512 | 100 | 100 | 100 | 99.02512 |
| 2 | 90.27653 | 98.72352 | 98.02468 | 41.34819 | 24.3568 |
|  | 89.14038 | 97.62354 | 97.63513 | 45.49762 | 22.84273 |
|  | 88.43682 | 99.22299 | 98.9255 | 44.82564 | 25.06925 |
| 4 | 83.52483 | 85.35283 | 94.35825 | 52.71215 | 22.34528 |
|  | 83.1413 | 81.96534 | 93.05432 | 49.93215 | 23.05163 |
|  | 80.03258 | 82.12545 | 93.92731 | 50.69146 | 21.09197 |
| 6 | 68.24776 | 77.35287 | 98.02589 | 48.11358 | 26.81243 |
|  | 71.20253 | 74.65932 | 97.15253 | 46.8124 | 22.05483 |
|  | 67.65283 | 78.17033 | 98.86749 | 47.66659 | 24.07809 |
| 8 | 74.04144 | 75.24682 | 90.75172 | 40.15315 | 20.39046 |
|  | 72.18725 | 71.02167 | 89.32851 | 37.54315 | 18.01857 |
|  | 72.03684 | 73.60688 | 94.0235 | 38.03584 | 21.82135 |
| 10 | 71.47578 | 71.82153 | 88.12145 | 31.45138 | 19.7397 |
|  | 69.05483 | 71.05397 | 87.01538 | 30.31528 | 18.1386 |
|  | 69.39325 | 75.101 | 90.56755 | 34.96922 | 17.01512 |

Table S11 Exploration of enzymatic properties (The Michaelis constant)

| C^-1^ | V^-1^(min·μmol^-1^) | | |
| --- | --- | --- | --- |
| (L·mol^-1^) | 1 | 2 | 3 |
| 20 | 1.84235 | 1.53258 | 1.9924 |
| 40 | 2.45682 | 2.23314 | 2.3242 |
| 60 | 3.17263 | 2.90235 | 3.0744 |
| 80 | 4.27364 | 4.02183 | 4.1057 |
| 100 | 4.93255 | 5.02735 | 4.8091 |

Table S12 the Repetitive availability of immobilized PGA

| Cycle number | Relative activity (%) | | |
| --- | --- | --- | --- |
|  | 1 | 2 | 3 |
| 1 | 98.05244 | 98.2153 | 100 |
| 2 | 76.91485 | 72.02451 | 74.49218 |
| 3 | 56.51252 | 57.96229 | 60.45112 |
| 4 | 45.44875 | 48.11224 | 41.0583 |
| 5 | 19.15101 | 18.0215 | 23.24182 |
